# Landiolol is effective and safe in paediatric supraventricular tachycardia: evidence from a European prospective multicentre open-label phase III study (LANDI-PED)

**DOI:** 10.1093/europace/euaf025

**Published:** 2025-02-14

**Authors:** Ina Michel-Behnke, Matthias Müller, Brigitte Stiller, Thomas Kriebel, Majed Kanaan, László Környei, Matthias Mai, Roman Gebauer, Jens Meier, Ferran Roses-Noguer, Martin Unger, Stefanie Schlager, Charu Charu, Christoph Klade, Kurt Krejcy, Jakob Ackerl, Günther Krumpl

**Affiliations:** Department of Paediatrics and Adolescent Medicine, Division of Paediatric Cardiology, Medical University of Vienna, General Hospital Vienna, Vienna, Austria; Department of Anaesthesiology and Intensive Care Medicine, Paediatric Heart Centre Giessen, University Hospital Giessen and Marburg GmbH, Giessen, Germany; Department of Congenital Heart Disease and Paediatric Cardiology, Faculty of Medicine, Heart Centre Freiburg University, University of Freiburg, Freiburg, Germany; Department of Paediatrics and Paediatric Cardiology, Westpfalz Hospital, Kaiserslautern, Germany; Department of Paediatric Cardiology, Children’s Heart Centre Aachen, University Hospital RWTH Aachen, Aachen, Germany; Gottsegen National Cardiovascular Centre, Paediatric Heart Centre, Budapest, Hungary; Paediatrics 3–Centre for Congenital Heart Defects Stuttgart, Paediatric Intensive Care, Pulmonology and Allergology, Stuttgart Hospital–Olgahospital, Stuttgart, Germany; Clinic for Paediatric Cardiology, Heart Centre Leipzig GmbH, Leipzig, Germany; Department of Anaesthesia and Intensive Care, Kepler University Clinic, Kepler University, Linz, Austria; Paediatric Cardiology Department, University Hospital Vall d’ Hebron, Barcelona, Spain; Honorary Paediatric EP Consultant, Royal Brompton and Harefield Hospital, London, UK and European Reference Network for Rare, Low Prevalence and Complex Diseases of the Heart (ERN GUARD-Heart); AOP Orphan Pharmaceuticals GmbH, Leopold-Ungar-Platz 2, 1190 Vienna, Austria; AOP Orphan Pharmaceuticals GmbH, Leopold-Ungar-Platz 2, 1190 Vienna, Austria; AOP Orphan Pharmaceuticals GmbH, Leopold-Ungar-Platz 2, 1190 Vienna, Austria; AOP Orphan Pharmaceuticals GmbH, Leopold-Ungar-Platz 2, 1190 Vienna, Austria; AOP Orphan Pharmaceuticals GmbH, Leopold-Ungar-Platz 2, 1190 Vienna, Austria; AOP Orphan Pharmaceuticals GmbH, Leopold-Ungar-Platz 2, 1190 Vienna, Austria; Board of Directors, AOP Health International Management AG, Ruggell, Liechtenstein

**Keywords:** Landiolol, Ultra-fast acting super-selective beta-blocker, Supraventricular tachycardia, Paediatrics, Heart rate, Sinus rhythm

## Abstract

**Aims:**

Landiolol, an ultra-fast acting super-selective beta-blocker, was investigated for the first time in Europe in a prospective clinical study for the management of supraventricular tachycardia (SVT) among paediatric patients.

**Methods and results:**

The LANDI-PED study was a prospective, multicentre, open-label, uncontrolled phase III study aiming to investigate the efficacy, safety, and pharmacokinetics (PK) of landiolol in paediatric patients. Sixty patients in surgical and non-surgical settings aged ≥1 day to <18 years with SVTs of various aetiologies received landiolol as a continuous intravenous infusion starting with 5 μg/kg/min titrated up to 40 μg/kg/min depending on heart rate (HR) reduction for up to a maximum of 24 h. The primary endpoint was restoration of normal sinus rhythm (NSR) within 210 min of infusion start. The primary endpoint was achieved in 15 (25.0%) patients. A total of 24 (40.0%) patients achieved a HR reduction of at least 20% within 210 min of landiolol infusion. A significant HR reduction was observed within minutes post-infusion, with a mean (±SD) reduction after 210 min of −13.2 (±11.5)% (*P* < 0.0001) in the overall population. By infusion end, 51.7% of patients achieved HR reduction of at least 20% from baseline and/or NSR conversion. The PK characteristics were consistent with the known profile of landiolol among adults. The most common adverse drug reaction was hypotension (10%).

**Conclusion:**

Landiolol is effective and safe in the treatment of SVTs in the paediatric population as demonstrated by reduction of HR and/or restoring NSR. Landiolol was well tolerated with no novel safety concerns reported.

**Clinical Trial Registration:**

EU Clinical Trial Register; EudraCT Number: 2015-001129-17.

## Structured Graphical Abstract

**Structured Graphical Abstract:**
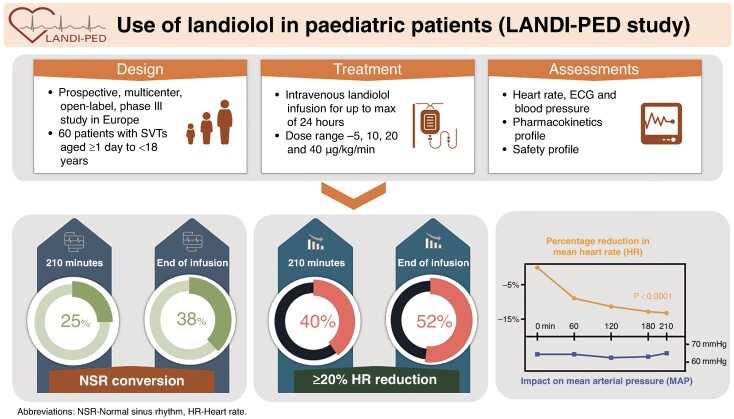
**Key question:** Can landiolol effectively and safely reduce heart rate and restore normal sinus rhythm in paediatric patients with sustained supraventricular tachycardia? **Key finding:** Landiolol effectively controlled supraventricular tachycardia in a significant proportion of patients, as shown by achievement of normal sinus rhythm and/or >20% heart rate reduction. Landiolol selectively reduced heart rate with rapid onset of action and minimal effect on blood pressure. **Take-home message:** Paediatric patients with supraventricular tachycardia can be successfully managed with landiolol, showing good tolerability and no novel safety concerns. Comparable pharmacokinetic profile with adults endorses its administration following the same dosage regimen.

**What’s new?**

- A safe and effective drug for the acute management of tachycardia in paediatric patients remains a key focus in paediatric cardiology and critical care.
- The LANDI-PED study is the first European prospective, multicentre clinical study investigating landiolol, an ultra-fast acting beta-blocker, for managing supraventricular tachycardia (SVT) in paediatric patients.
- Landiolol showed efficacy in treating SVTs across diverse aetiologies among children of all ages. The fast onset and minimal impact on blood pressure support its role in acute heart rate control where haemodynamic stability is essential.
- Landiolol was well tolerated, with no new safety concerns.
- Pharmacokinetic characteristics of landiolol in paediatric patients were consistent with those in adults.
- The study demonstrated landiolol’s potential in managing SVT in paediatric patients, aligning with its established therapeutic role in adults.

## Introduction

Supraventricular tachycardia (SVT) in paediatric patients presents considerable diagnostic and therapeutic challenges as they can potentially cause haemodynamic instability and compromised cardiac function.^1–7^ Supraventricular tachycardias encompass a spectrum of conditions with and without rhythm disturbances, including inappropriate sinus tachycardia (IST), junctional ectopic tachycardia (JET), atrial flutter (AF), atrial fibrillation (AFib), focal atrial tachycardia (FAT), atrioventricular nodal re-entrant tachycardia (AVNRT), and atrioventricular re-entrant tachycardia (AVRT), with each subtype requiring a tailored approach.^5,8^ Paediatric arrhythmia management remains challenging due to limited validation of antiarrhythmic agents caused by fewer studies conducted in this population compared to adults. While advancements in diagnostics and therapies have emerged,^9^ the need for approved pharmacological agents remains unmet.^9^

Beta-blockers are widely acknowledged as standard treatment for acute tachycardia due to their ability to block beta-adrenergic receptors, those having a rapid onset and short duration of action being particularly suitable for situations requiring immediate heart rate (HR) control.^10–12^ Landiolol, an ultra-fast-acting, super-selective beta-blocker, has been used for decades to treat tachyarrhythmias in adults, with numerous studies demonstrating its efficacy in treating SVT in this population.^13–23^ Compared to other beta-blockers, landiolol has a shorter half-life of approximately 3–4 min due to its rapid metabolism by plasma esterases and low volume of distribution.^24^ Additionally, it demonstrates substantially higher β1-selectivity (β1/β2 = 255) compared to esmolol (β1/β2 = 33) or propranolol (β1/β2 = 0.68), thereby exhibiting a weaker negative inotropic effect but a pronounced negative chronotropic potential.^21,25–28^ The limited impact of landiolol on inotropy can be attributed to partial receptor occupancy and its minimal effect on membrane currents, including L-type calcium channels.^27,29–32^ These properties along with the absence of chaperoning and rebound effects makes landiolol a preferable choice over other beta-blockers, including esmolol, in scenarios requiring fine-tuned heart rate control with minimal risk of haemodynamic instability.^27,29^

Landiolol received its initial approval in Japan in 2002 in the adult population under the brand name Onoact^®^, followed by approval in the European Union in 2016 for the treatment of SVTs under the trade names Rapibloc^®^, Raploc^®^, Landiobloc^®^, and Runrapiq^®^.^33^ The current European guidelines recommend the use of beta-blockers, such as landiolol, as first-line drugs to control HR in AFib patients^34^ as well as for the acute management of paroxysmal SVTs when vagal manoeuvres and adenosine are ineffective.^34,35^

Numerous case reports^36–40^ and several retrospective, observational studies^41–45^ have reported the use of landiolol in paediatric patients, primarily within the Asian population. According to these observations, landiolol exhibited good tolerability and high clinical efficacy for treating tachyarrhythmia of various aetiologies, as demonstrated by a positive response in more than 70% of patients, indicated by a conversion to sinus rhythm and/or HR reduction.^41–45^ A recent prospective phase II/III registration study (HEARTFUL study) in Japanese paediatric patients demonstrated that landiolol reduced HR by at least 20% or terminated tachycardia within 2 h in 48.0% of patients,^46^ leading to its paediatric approval in Japan in 2022 for treating tachyarrhythmia (SVT, AFib, and AF) in patients with low cardiac function.^47^

In Europe, no antiarrhythmic agent apart from adenosine for AVRT and AVNRT is currently approved for the acute management of SVTs in paediatric patients. There is no definitive first-line treatment of SVTs in children, and drug effectiveness varies significantly across different SVT types.^11^ Despite a favourable tolerability and efficacy profile of landiolol shown by the available studies, the major limitations for the use of landiolol are the absence of prospective studies outside Japan; monocentric, retrospective study designs; restricted patient populations and arrhythmia types; and small sample sizes. Therefore, the LANDI-PED study aimed to investigate the efficacy and safety of landiolol in treatment of SVTs in the paediatric population for the first time in Europe in a prospective, multicentre, open-label, uncontrolled phase III study.

## Methods

### Study design and ethics

This was a prospective, multicentre, open-label, uncontrolled phase III study conducted at 10 hospitals in four European countries (Austria, Germany, Hungary, and Spain). Participating centres are listed in Supplementary material online, *Table S1* (see Supplementary material). The study was performed as specified in the Paediatric Investigational Plan (PIP) approved by the European Medicines Agency (EMA)/Paediatric Committee (PDCO) and was conducted in compliance with the Declaration of Helsinki, ICH Good Clinical Practice (GCP) guidelines, and local laws after approval of the study protocol by the independent Ethics Committee at each participating site. The study was overseen by an independent Data Safety Monitoring Board. The study was registered at EudraCT (2015-001129-17).

### Patient population

The inclusion and exclusion criteria are outlined in Supplementary material online, *Table S2*. Briefly, paediatric patients aged ≥1 day up to 18 years who had undergone or were scheduled for surgery (cardiac and non-cardiac procedures), as well as non-surgical patients weighing at least 2.5 kg and who had sustained SVT lasting longer than 1 min, were included in the study. Patients with AVNRT and AVRT were enrolled only if they were either refractory to adenosine, had relapsed after treatment with adenosine, or had contraindications to adenosine. Key exclusion criteria were acute cardiogenic shock, ventricular tachycardia, sick sinus syndrome without possibility for cardiac pacing, clinically significant bradycardia, known pulmonary hypertension, and decompensated heart failure. Recruitment was stratified by age into two age groups (≥1 day to <2 years and ≥2 years to <18 years).

### Treatment

Lyophilized landiolol hydrochloride was reconstituted in 50 mL of 0.9% NaCl to a 6 mg/mL concentration (i.e. 300 mg/50 mL) just before administration. If required, reconstituted landiolol was further diluted to a final concentration of 0.6 mg/mL. Patients received a continuous intravenous infusion of landiolol hydrochloride at a starting dose of 5 µg/kg/min. Dosing was gradually increased every 30 min or every 10 min in later protocol versions to the next higher dose levels of 10 µg/kg/min, 20 µg/kg/min, and 40 µg/kg/min (see Supplementary material online, *Figure S1*). Once a HR reduction of at least 20% was achieved, this dose was maintained until minute 210. If a higher HR reduction was medically indicated and deemed safe, further dose escalation (up to a maximum of 40 µg/kg/min) could be performed at the investigator’s discretion. Landiolol infusion could be extended beyond 210 min up to a maximum of 24 h (prolongation phase), provided it was medically justified and considered safe and effective by the investigator and no suitable alternative treatments were available.

Concomitant administration of antiarrhythmic agents Class I–V with landiolol for the purpose of controlling HR or terminating the SVT was prohibited, except for overlapping administration with landiolol close to infusion end as a part of switch therapy. In case of transition to oral therapy, the dose of landiolol was to be reduced to 50% 30 min after initiation of oral therapy followed by termination of landiolol infusion 30 min later. The type (e.g. any alternative antiarrhythmic therapy), dose, and duration of subsequent treatment or rescue therapy post-landiolol infusion were at the discretion of investigators.

### Assessments

All measurements and assessments are listed in detail in the study flowchart in Supplementary material online, *Figure S2*. The total study duration was 8 days per patient consisting of screening, infusion, optional prolongation phase and follow-up visits scheduled at 1 h, 24 h, and 7 days post-landiolol infusion. Haemodynamic parameters (HR, systolic and diastolic BP) were recorded at 5 min intervals during the first 30 min of landiolol infusion, at 10 min intervals between minute 30 and minute 210, at 4 h intervals during the prolongation phase, and at the end of infusion with additional measurements during follow-up visits. Twelve-lead electrocardiogram (ECG) was performed regularly throughout the infusion period and at every follow-up visit. Further HR, BP, and ECG measurements were performed upon occurrence of relevant adverse events (AEs), dose adjustments, conversion to normal sinus rhythm, or whenever deemed necessary by the investigator. Laboratory assessments for haematology (complete blood count and coagulation profile), clinical chemistry (hepatic, renal function and electrolytes), and selected enzymes were performed at screening and follow-up visits on 24 h and Day 7. Safety assessments were also performed for seriousness and possible causal relationship with landiolol. Serious AEs (SAEs) were defined as an undesirable sign, symptom, or medical condition that results in death; is life-threatening; requires inpatient hospitalization or prolongation of existing hospitalization; results in persistent or significant disability or incapacity; is a congenital anomaly or birth defect; or is medically significant. The causal relationship between AEs and landiolol was determined by the investigator’s medical judgment, considering pattern of reaction, temporal relationship, concomitant medications, diseases, and medical history. For PK assessment, blood samples were collected at specified intervals as indicated in Supplementary material online, *Figure S2*. Landiolol was quantified with the validated method as described earlier.^48^

### Study endpoints

The primary endpoint was the percentage of patients converting to normal sinus rhythm within 210 min of the commencement of landiolol infusion. For IST patients, normal sinus rhythm was defined as maintenance of sinus rhythm and reduction of HR to normal range as assessed by the investigator. Secondary efficacy endpoints included percentage change in HR during the infusion, number of patients who achieved at least 20% reduction in HR at 210 min as compared to baseline, relative and cumulative HR reduction rates at each dosing level, number of patients who achieved either NSR conversion or HR reduction during prolongation phase, percentage of patients with sustained response for more than 24 h and 7 days post-infusion, relationship between HR change and patient’s age, percentage of patients with prolonged infusion period and duration of prolongation, time required to achieve >20% HR reduction, and NSR conversion. Half-life, total body clearance, and volume of distribution of landiolol were pharmacokinetic (PK) endpoints. Safety endpoints evaluated the incidence and severity of AEs including SAEs and percentage of patients requiring discontinuation or dose reduction of landiolol due to safety concerns.

### Statistical analysis

This study did not test a formal hypothesis. The decision on participant numbers was based on eligibility criteria and regulatory guidance from PDCO to ensure comprehensive efficacy and safety evaluation. Initially, the goal was to enrol 120 patients. However, due to challenges in achieving this sample size within the designated timeframe, the target was adjusted to 60 patients in 2021, with approval from EMA/PDCO (Supplementary material online, *Methods*). A subgroup analysis was conducted by age and *post hoc* on selected patient characteristics including sex, surgical status (non-surgical, peri-operative, post-operative), SVT type at baseline, baseline use of catecholamines and inotropes, and prior use of beta-blockers. Statistical analysis was performed using Statistical Analysis System (SAS^®^) software, version 9.4 (SAS Institute, Cary, NC, USA). Categorical variables were summarized with absolute frequencies and percentages. Asymptotic Wald’s 95% confidence intervals (CIs) were calculated as precision estimates. Continuous variables were summarized using descriptive statistics (mean, standard deviation, median, 1st and 3rd quartile, and range) and, wherever relevant, two-sided 95% CIs were presented as precision estimates. Difference between medians was evaluated using one-sample Wilcoxon signed-rank test. The PK parameters were calculated using a non-compartmental analysis (Phoenix^®^ WinNonlin software, version 8.4; Pharsight Corporation, USA).

## Results

### Patient disposition and baseline characteristics

**Figure 1:**
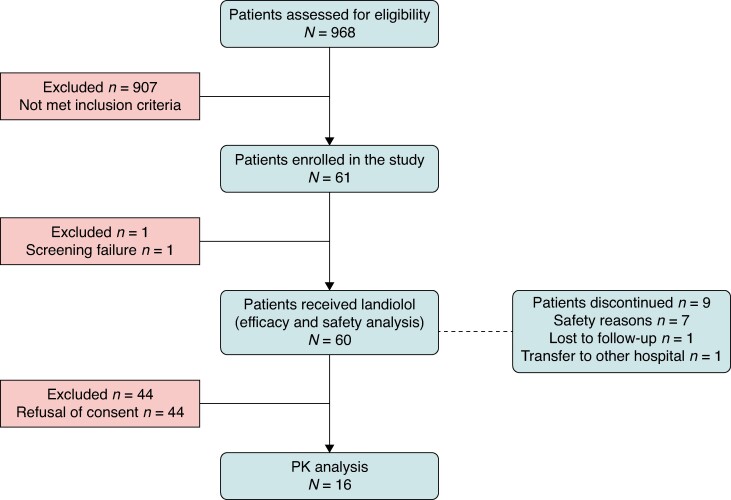
Patient enrolment flow in LANDI-PED study.

Nine hundred and sixty-eight patients aged ≥1day to <18 years who were at risk of developing SVT during the hospital stay were screened for potential eligibility in the study between November 2018 and May 2023. Of these, 907 patients did not experience an episode of SVT or met inclusion criteria. A total of 61 patients were enrolled in the study, reflecting a similar enrolment rate seen in other paediatric studies.^46^ One patient was excluded before landiolol administration due to screening failure. In total, 60 patients received treatment with landiolol and were included in the efficacy and safety analysis. The study was prematurely discontinued in nine patients, out of which seven were due to safety reasons and two were lost to follow-up after 24 h of landiolol infusion end. For PK analysis, 16 patients gave consent for sampling (*Figure 1*).

Baseline demographics and clinical characteristics of patients are depicted in *Table 1*. The study included 34 (56.7%) females and 26 (43.3%) males with 40 (66.7%) patients aged from ≥1 day to under 2 years and 20 (33.3%) patients aged from 2 years to less than 18 years. The mean (±SD) age was 6.2 (±5.1) months for those aged less than 2 years and 7.8 (±4.4) years for those aged 2 years to 18 years with a mean (±SD) body weight at baseline of 14.2 (±17.3) kg for the overall population. The mean (±SD) heart rate was 164.2 (±28.4) b.p.m. and mean (±SD) arterial pressure was 64.4 (±12.7) mmHg, indicating that the patients were generally haemodynamically stable. The study predominantly comprised patients of white ethnicity (90.0%).

**Table 1:** Baseline characteristics of patients

Patient characteristics	*N* = 60
Gender, *n* (%)	
Female	34 (56.7%)
Male	26 (43.3%)
Age	
≥1 day to <2 years, *n* (%)	40 (66.7%)
Mean (±SD), in months	6.2 (±5.1)
Median (min–max)	5.0 (0–22)
>2 years to <18 years, *n* (%)	20 (33.3%)
Mean (±SD), in years	7.8 (±4.4)
Median (min–max)	5.6 (3–16)
Weight (kg), mean (±SD)	14.2 (±17.3)
Heart rate (b.p.m.), mean (±SD)	164.2 (±28.4)
Mean arterial pressure (mmHg), mean (±SD)	64.4 (±12.7)
Systolic blood pressure (mmHg), mean (±SD)	88.7 (±19.9)
Diastolic blood pressure (mmHg), mean (±SD)	52.3 (±10.5)
Ethnicity, *n* (%)	
White	54 (90.0%)
Asian	1 (1.7%)
Black or African American	1 (1.7%)
Other	4 (6.7%)
Underlying heart diseases, *n* (%)	54 (90.0%)
Prior cardiac surgery, *n* (%)	
Prior cardiac surgery within last week	24 (40.0%)
Prior cardiac surgery any time in the past	31 (51.7%)
Patients’ status with respect to surgery, *n* (%)	
Non-surgical	17 (28.3%)
Peri-operative	14 (23.3%)
Post-operative	29 (48.3%)
SVT type at baseline, *n* (%)	
IST	30 (50.0%)
JET	12 (20.0%)
FAT	8 (13.3%)
AVRT	7 (11.7%)
Other	3 (5.0%)
Time since SVT start (min)	
Mean (±SD)	1246.2 (±4072.9)
Median (Q1–Q3)	38.0 (0.0–490.0)
Prior therapies^a^, *n* (%)	
Prior use of beta-blockers	14 (23.3%)
Catecholamines at baseline	21 (35.0%)
Inotropes at baseline	32 (53.3%)

Data are represented as number (percentage), mean (±SD), or median with minimum–maximum or Q1–Q3.

SVT, supraventricular tachycardia; IST, inappropriate sinus tachycardia; JET, junctional ectopic tachycardia; FAT, focal atrial tachycardia; AVRT, atrioventricular re-entrant tachycardia.

^a^Recorded use of beta-blockers that ended within 7 days before landiolol administration. The use of catecholamines includes norepinephrine, epinephrine, and phenylephrine and of inotropes milrinone, levosimendan, and dobutamine.

Underlying cardiac disease was noted in 54 (90.0%) patients and 24 (40.0%) patients had undergone cardiac surgery within the week before treatment with landiolol. History of cardiac surgery at any point in the past was reported in 31 (51.7%) patients. In terms of surgical status, 17 (28.3%) patients belonged to non-surgical category, while 14 (23.3%) patients were in the peri-operative period and 29 (48.3%) were post-operative patients. Regarding the aetiology of SVT at baseline, most patients had IST (50.0%), followed by JET (20.0%), FAT (13.3%), or AVRT (11.7%), and for three patients (5.0%), SVT types were classified as ‘other’ (two cases of atrial ectopic tachycardia and one case of multifocal SVT). The distribution of patients per SVT subtypes and surgical status is shown in Supplementary material online, *Table S3*. The median time since the onset of SVT was 38 (Q1–Q3:0–490.0) minutes. A total of 23.3% of patients had a history of beta-blocker use. At baseline, 35.0% of patients were administered catecholamines, 53.3% received inotropes, and a comparable percentage had concomitant use of catecholamines and inotropes (see Supplementary material online, *Table S4*).

### Efficacy

The primary endpoint, conversion to NSR within 210 min of the commencement of landiolol infusion, was achieved in 15 (25.0%) patients (*Table 2*). A total of 17 patients (28.3%) met the criteria for entering the prolongation phase and received landiolol for an additional duration of 16.9 (±5.2) hours. During that time, eight patients achieved NSR conversion, resulting in a total of 23 (38.3%) patients with normal sinus rhythm by the end of landiolol infusion. A HR reduction of at least 20% was achieved in 24 (40.0%) patients within 210 min and in 7 additional patients during the prolongation phase, totalling 31 (51.7%) patients by the end of infusion. The endpoint of either conversion to NSR or at least 20% HR reduction within 210 min was met by 24 (40.0%) patients and 7 more patients achieved this endpoint during the prolongation phase, resulting in a total of 31 (51.7%) patients by the end of infusion. The reduction in HR was significant with a mean relative change (±SD) from baseline of −13.2 (±11.5)% at 210 min (*P* < 0.0001) and −16.0 (±15.6)% at the end of infusion (*P* < 0.0001). The median time to achieve NSR conversion was 169 min (Q1–Q3: 60–450; min–max: 37–1344) (see Supplementary material online, *Figure S3*) and the median time to reach the target HR reduction (≥20%) was 107 min (Q1–Q3: 40–170; min–max: 10–1344) (see Supplementary material online, *Figure S4*).

**Table 2:** Primary and selected secondary efficacy analysis

Treatment phase	Time point	Patients with NSR conversion	Patients with ≥20% HR reduction	Patients with either NSR conversion or ≥20% HR reduction	Mean relative HR change from baseline
Landiolol infusion phase	Up to 210 min	15 (25.0%) (95% CI: 14.0; 35.9)	24 (40.0%) (95% CI: 27.6; 52.4)	24 (40.0%) (95% CI: 27.6; 52.4)	−13.2% (±11.5) (95% CI: −16.7; −9.8) *P* < 0.0001
	Up to infusion end (max 24 h)	23 (38.3%) (95% CI: 26.0; 50.6)	31 (51.7%) (95% CI: 39.0; 64.3)	31 (51.7%) (95% CI: 39.0; 64.3)	−16.0% (±15.6) (95% CI: −20.0; −11.9) *P* < 0.0001
Follow-up phase	Up to 24 h post-infusion end	35 (58.3%) (95% CI: 45.9; 70.8)	37 (61.7%) (95% CI: 49.4; 74.0)	44 (73.3%) (95% CI: 62.1; 84.5)	−21.6% (±17.5) (95% CI: −26.2; −17.0) *P* < 0.0001
	Up to 7 days post-infusion end	48 (80.0%) (95% CI: 69.9; 90.1)	41 (68.3%) (95% CI: 56.6; 80.1)	52 (86.7%) (95% CI: 78.1; 95.3)	−23.6% (±16.9) (95% CI: −28.1; −19.1) *P* < 0.0001

Data are represented as cumulative response rates, *n* (percentage), and as mean (±SD) relative HR change from baseline and 95% CIs.

NSR, normal sinus rhythm; HR, heart rate.

During the post-infusion follow-up phase, wherein alternative pharmacological and/or other therapies could be used, normal sinus rhythm was achieved in an additional 12 patients within the first 24 h and 13 patients by 7 days, resulting in 80% of patients attaining a normal sinus rhythm. Similarly, within 24 h and 7 days after infusion end, an additional 6 and 10 patients, respectively, achieved a decrease in HR exceeding 20%, raising the cumulative total to 41 (68.3%) patients. Overall, 7 days after infusion end, 52 (86.7%) patients achieved either at least 20% HR reduction or normal sinus rhythm. A continuous HR reduction was observed during the follow-up phase with −21.6 (±17.5)% and −23.6 (±16.9)% reduction at 24 h (*P* < 0.0001) and 7 days after infusion end (*P* < 0.0001), respectively.

**Figure 2:**
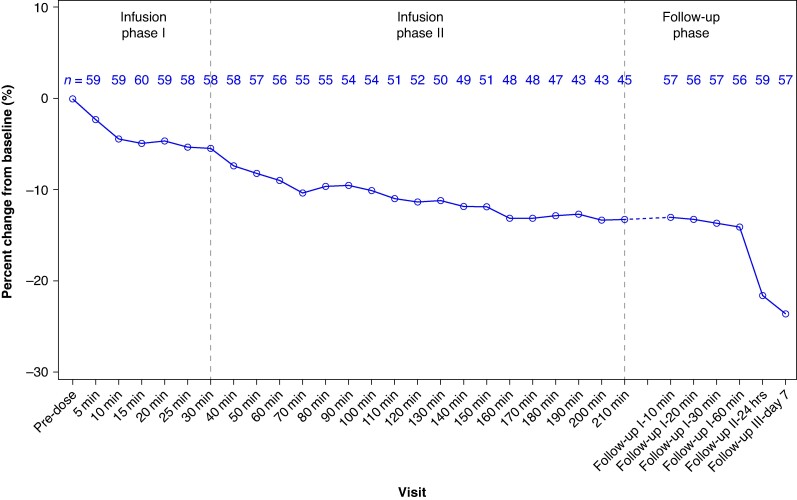
Relative reduction in HR from baseline to 210 min and during follow-up phase. The reduction in mean percentage HR was statistically significant at all time points from minute 5 onwards until infusion end.

Heart rate showed a swift response to landiolol resulting in a significant decrease within minutes of starting the infusion as shown in *Figure 2*. A mean relative reduction in HR from baseline of −2.3 (±6.1)% was observed at minute 5 (*P* < 0.0001), which further increased to −5.5 (±7.4)% at minute 30. Heart rate continued to decrease further to −8.9 (±10.9)% at minute 60 and −11.3 (±12.6)% at minute 120. Patients who achieved >20% HR reduction showed early signs of landiolol efficacy with swift and consistent reduction in HR from minute 5 onwards. For patients with prolonged infusion beyond 210 min, a continuous HR reduction was achieved with relative reductions of −24.6 (±13.4)% at 450 min, −27.7 (±16.3)% at 930 min, and −35.8 (±15.2)% at 1410 min (see Supplementary material online, *Figure S5*).

**Figure 3:**
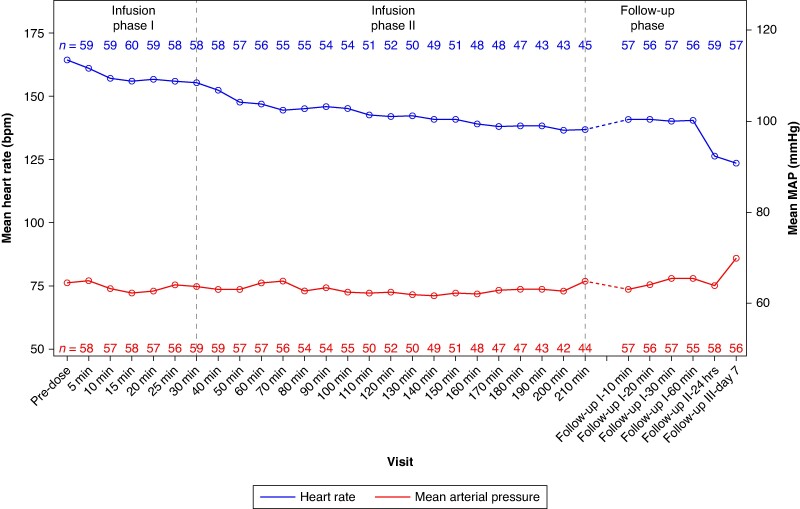
Absolute HR (left *y*-axis; top line) and mean arterial pressure (MAP) (right *y*-axis; bottom line) from baseline to 210 min and during follow-up phase. The mean MAP was overall stable, while the absolute HR reduction was statistically significant at all time points from minute 5 onwards until infusion end.

Mean arterial pressure (MAP) remained stable throughout the study period, with baseline values of 64.4 (±12.7) mmHg and 65.0 (±11.8) mmHg at 210 min. The mean absolute HR at baseline was 164.2 (±28.4) b.p.m., which significantly reduced to 136.8 (±25.6) b.p.m. at 210 min (*P* < 0.0001) (*Figure 3*).

The treatment duration varied between patients with the shortest treatment duration of 20 min to a maximum of 1440 min and a mean administration time of 476.9 (±504.4) minutes. The mean total dose of landiolol administered over the whole treatment period was 14.5 (±17.9) mg/kg, with a median dose 6.1 mg/kg, and 56.8 mg/kg as the maximum total administered dose. The highest administered doses were spanning the full dosing range of 5–40 µg/kg/min with a mean and median of 34.9 and 40 µg/kg/min, respectively (see Supplementary material online, *Table S5*).

The response rates increased with higher doses of landiolol (see Supplementary material online, *Table S6*). Among the 31 patients who achieved ≥20% reduction in HR at any time point during infusion, 20 (64.5%) patients reached this endpoint at the dose of 40 µg/kg/min, and similarly, of the 23 patients who converted to NSR during infusion, 19 (82.6%) patients achieved this endpoint at the dose of 40 µg/kg/min.

### Safety

A total of 46 AEs were reported in 35 (58.3%) patients (*Table 3*). Among these, 6 events in 6 (10.0%) patients were classified as drug-related according to the investigator, all of which were cases of hypotension. Among these drug-related hypotension cases, three were also considered related to the underlying SVT, due to the prolonged state of tachycardia and subsequent decompensation. In all instances of hypotension, blood pressure dropped transiently and recovered upon discontinuation or dose reduction of landiolol. Serious AEs occurred in four (6.7%) patients, with only one event of hypotension in one (1.7%) patient rated as drug-related. Five patients (8.3%) discontinued landiolol and one (1.7%) had a dose reduction due to AEs, all of which were cases of hypotension linked to a causal relationship with landiolol. No death was reported in the LANDI-PED study. Landiolol was well tolerated even at higher doses, with no major safety risks.

**Table 3:** Safety profile of landiolol

AEs	Number of AEs	Number of patients with AEs	
	Total	Total	Related to study drug
All	46	35 (58.3%)	6 (10.0%)
Serious	4	4 (6.7%)	1 (1.7%)
Leading to discontinuation	8	7 (11.7%)	5 (8.3%)
Leading to dose reduction	1	1 (1.7%)	1 (1.7%)
By MedDRA preferred term			
Hypotension	8	8 (13.3%)	6 (10.0%)
Pleural effusion	5	5 (8.3%)	0 (0.0%)
Hypokalaemia	2	2 (3.3%)	0 (0.0%)
Pulmonary hypoperfusion	1	1 (1.7%)	0 (0.0%)
Stridor	1	1 (1.7%)	0 (0.0%)
Infusion site erythema	1	1 (1.7%)	0 (0.0%)
Cardiac tamponade	1	1 (1.7%)	0 (0.0%)
Aortic valve incompetence	1	1 (1.7%)	0 (0.0%)
Ejection fraction decreased	1	1 (1.7%)	0 (0.0%)
Prolonged QRS complex on electrocardiogram	1	1 (1.7%)	0 (0.0%)
Pyrexia	1	1 (1.7%)	0 (0.0%)
Abdominal pain	1	1 (1.7%)	0 (0.0%)
Hypertonia	1	1 (1.7%)	0 (0.0%)

Occurrence of AEs as number of events and number (percentage) of patients, with drug-related AEs (according to investigators) shown separately.

AEs, adverse event; MedDRA, Medical Dictionary for Regulatory Affairs.

### Subgroup analysis

A *post hoc* subgroup analysis at the 210 min time point revealed variability in response rates based on demographic characteristics and clinical conditions (*Table 4*). In general, across all subgroups higher number of patients achieved ≥20% HR reduction as compared to NSR conversion. Older patients (ages >2 years to <18 years), males, and those not undergoing surgery had higher response rates for both endpoints. Detailed analysis of changes in HR by patient’s age was performed and is outlined in Supplementary material online, *Figure S6*. Among SVT types, AVRT patients responded best to landiolol with NSR conversion and HR reduction in 57.1% and 85.7% of patients respectively, while the lowest response rates were seen in JET patients (8.3% in NSR conversion and 25.0% in HR reduction). The response rates were relatively consistent across patients with and without prior use of beta-blockers. However, patients who did not receive inotropes or catecholamines at baseline demonstrated better response to landiolol than those who received them.

**Table 4:** Subgroup analysis for response to landiolol at 210 min

Subgroups	Patients with NSR conversion at 210 min *n*/*N* (%)	Patients with ≥20% HR reduction at 210 min *n*/*N* (%)
Age		
≥1 day to <2 years	7/40 (17.5)	14/40 (35.0)
>2 years to <18 years	8/20 (40.0)	10/20 (50.0)
Gender		
Female	5/34 (14.7)	11/34 (32.4)
Male	10/26 (38.5)	13/26 (50.0)
Patients’ status with respect to surgery		
Non-surgical	8/17 (47.1)	11/17 (64.7)
Peri-operative	3/14 (21.4)	4/14 (28.6)
Post-operative	4/29 (13.8)	9/29 (31.0)
SVT type at baseline		
IST	8/30 (26.7)	10/30 (33.3)
JET	1/12 (8.3)	3/12 (25.0)
FAT	2/8 (25.0)	4/8 (50.0)
AVRT	4/7 (57.1)	6/7 (85.7)
Others	0/3 (0.0)	1/3 (33.3)
Prior use of beta-blockers^a^		
Yes	4/14 (28.6)	6/14 (42.9)
No	11/46 (23.9)	18/46 (39.1)
Catecholamines at baseline^b^		
Yes	4/21 (19.1)	6/21 (28.6)
No	11/39 (28.2)	18/39 (46.2)
Inotropes at baseline^c^		
Yes	4/32 (12.5)	7/32 (21.9)
No	11/28 (39.3)	17/28 (60.7)

Data represented as number (percentage).

NSR, normal sinus rhythm; HR, heart rate; SVT, supraventricular tachycardia; IST, inappropriate sinus tachycardia; JET, junctional ectopic tachycardia; FAT, focal atrial tachycardia; AVRT, atrioventricular re-entrant tachycardia.

^a^Recorded use of beta-blockers that ended within 7 days before landiolol administration.

^b^The use of catecholamines includes norepinephrine, epinephrine, and phenylephrine.

^c^The use of inotropes includes milrinone, levosimendan, and dobutamine.

### Pharmacokinetics

A total of 16 patients consented to the PK sampling. The steady state mean plasma concentrations of landiolol were 0.2 µg/mL, 0.5 µg/mL, and 0.6 µg/mL, respectively, for administered doses of 10 µg/kg/min, 20 µg/kg/min, and 40 µg/kg/min. After continuous landiolol infusion of 5–40 µg/kg/min, the median half-life of landiolol was 4.3 min and median total body clearance was 46 mL/min/kg. The median volume of distribution was 246 mL/kg.

## Discussion

In this first prospective, multicentre, phase III study in Europe, we present substantiative evidence on the efficacy, safety, and PK of landiolol as first-line treatment of SVTs or second-line therapy in AVRT/AVNRT patients covering a broad age range within the paediatric population. In this study, landiolol effectively controlled SVTs of various aetiologies in a significant proportion of paediatric patients. Over 50% of patients achieved either at least 20% reduction in HR and/or sinus rhythm conversion during landiolol treatment, which is comparable to findings observed in adults.^23^ As expected, due to its PK profile, we observed a rapid HR lowering effect within a few minutes of the start of infusion and a dose-dependent decrease in HR throughout the treatment period. Additional responders in the optional 24 h prolongation phase emphasize the rationale of continuing landiolol infusion beyond the acute treatment phase in patients showing initial heart rate reduction and clinical improvement. Notably, only an additional 17% of patients achieved at least 20% HR reduction within 7 days after the end of landiolol infusion, even with available standard treatment or alternative therapy. This indicates that landiolol treatment was effective in the vast majority of patients requiring a reduction of HR > 20%. Consistent with previous clinical studies conducted in both adult and paediatric populations, landiolol therapy had a limited impact on blood pressure, with patients maintaining stable blood pressure throughout the infusion.^41,45,49^ This finding demonstrates the ability of landiolol to selectively decrease heart rate without jeopardizing overall haemodynamic stability, a balance that is important in critical care settings and, particularly in vulnerable populations, such as paediatric patients.

*Post hoc* subgroup analysis identified landiolol as most effective in AVRT patients who, according to the inclusion criteria, had relapsed or were refractory to or contraindicated for adenosine, highlighting landiolol’s potential as an effective treatment option for refractory and/or recurrent SVT. Junctional ectopic tachycardia patients exhibited the lowest response rates, which contrasts with other published studies, reporting a more favourable response of JET patients to landiolol.^41–44,46^ However, direct comparison with these findings may be difficult as response to treatment varies significantly by JET subtype with some subtypes more resistant to interventions and prone to haemodynamic instability due to complex pathophysiological mechanisms.^50^ Furthermore, a substantial number of JET cases occur early in the post-operative period following congenital heart diseases surgery and often resolve spontaneously within a few days, making cross study comparison difficult.^11,51^

The use of landiolol was found to be safe and well tolerated. The most frequently reported AE was hypotension. All cases were transient and generally recovered promptly upon dose reduction or discontinuation of landiolol, demonstrating the benefit of its ultra-short half-life. Half of the hypotension cases occurred after prolonged states of tachycardia and were considered related to the underlying SVT, complicating the determination of a direct causal relationship with landiolol. Consistent to our findings, other studies also identified hypotension as predominant AE, as reported in 5.8–12.8% of patients.^13,52,53^ Notably, the incidence of hypotension was lower than the 20% reported in the HEARTFUL study,^46^ which used a lower dose range (1 to 10 μg/kg/min) compared to our study suggesting that the dose of landiolol does not necessarily correlate with the occurrence of AEs and higher doses do not pose an additional safety risk to patients. Considering the well-known safety profile of landiolol,^54–56^ there were no novel AEs reported in this study.

Guideline-recommended antiarrhythmic drugs for acute management of SVTs in paediatric patients include adenosine, digoxin, verapamil, flecainide, propafenone, and amiodarone.^11^ Adenosine’s short half-life makes it a valuable option in acute management of paroxysmal SVTs though it may lead to rapid recurrence, requiring antiarrhythmic drugs with longer half-life.^11^ Amiodarone has demonstrated efficacy, particularly in managing JET but conversion to sinus rhythm can take hours^42^ and carries significant extracardiac safety risks due to high incidence of thyroid, pulmonary, liver, skin, ocular, and neurologic toxicities.^11,57^ Optimal results are often achieved when these medications are used in conjunction with other drugs, such as beta-blockers and calcium channel blockers.^42^ Other recommended antiarrhythmic treatment options depending upon the SVT type and cardiac function are propafenone, flecainide, and verapamil, the latter being contraindicated in pre-excitation.^11^ Esmolol, even though not guideline-recommended for paediatric use to treat SVT, is used off-label in children. Some clinical studies have reported the use of esmolol in treating SVTs and hypertension in paediatric patients,^3,58,59^ but its hypotensive effect limits its wider use in this population.

The results from the LANDI-PED study add to the existing knowledge about the use of landiolol in the paediatric population.^44–46^ The HEARTFUL study reported that 48% of patients exhibited a reduction in HR of ≥20% from baseline or termination of tachycardia at 2 h with landiolol,^46^ which is comparable to the 40% response rate observed in the current study for the endpoint of NSR conversion and/or at least a 20% HR reduction. The LANDI-NEONATE study reported a 91.9% response rate within 24 h (with response defined as reduction of HR to normal levels) and a median time to achieve ≥20% HR reduction of 2.9 h, which is longer than the observed median time of 1.8 h in the present study as well as in previously published studies.^43^ It is important to note that previous studies not only differed in exact endpoint definitions but also in terms of study design, patient populations, and landiolol dosing, making direct comparisons between studies difficult. While the HEARTFUL study included patients aged ≥3 months to <15 years with cardiac dysfunction and various types of SVTs, the LANDI-NEONATE study focussed on critically ill neonates, including preterm infants and those with cardiac dysfunction and/or severe postnatal complications like pulmonary hypertension, aiming to improve overall haemodynamic stability. Consistent with dosing strategies for adults, lower starting doses (1–10 μg/kg/min) were used in both studies for patients with reduced ventricular ejection fraction (25–50% before the occurrence of arrhythmia), patients using cardiopulmonary assist devices and preterm children with cardiac dysfunction and/or pulmonary hypertension.^45,46^ Our study generally followed the dosing recommendation for adults without cardiac dysfunction^33^ (ranging from 10 to 40 μg/kg/min) except for a lower starting dose of 5 μg/kg/min. While some patients achieved target HR reduction at that initial dose, sinus rhythm conversion and HR response rates increased with higher doses of landiolol, with the dose level of 40 μg/kg/min proving to be most effective.

The comparable PK profile of landiolol in adults and children further supports the reasoning that dosage range in paediatric population should be similar as in adults. The half-life in the paediatric population was 4.3 min, which aligns with data from adults with reported values ranging between 4 and 4.5 min.^27,60^ Similarly, the total body clearance was comparable with the reported values of 43 mL/min/kg in another paediatric study^46^ and 53 mL/min/kg reported among healthy adults.^48^

Certain limitations of this study should be stated. The patient population was heterogeneous, consisting of paediatric patients of all ages, in a multitude of hospital settings and with different SVT subtypes (including adenosine pre-treated AVRT patients). However, limited racial and ethnic diversity in the study cohort may affect the generalizability of the findings to more diverse populations. While the primary endpoint was conversion to normal sinus rhythm, landiolol dosing was determined based on the HR response. Furthermore, the protocol followed a rigid dosing schedule that did not allow for a flexible individual dosing approach based on patient response, potentially limiting the advantages of a titratable ultra-fast acting agent. The PK results are limited by small sample size, variation in duration of landiolol infusion, and dose at the time of sample collection.

In conclusion, the LANDI-PED study validates the safety and efficacy of landiolol in treatment of SVTs across various aetiologies among children of all ages. Pharmacokinetic parameters were consistent with the established PK profile in adults confirming the ultra-short half-life of landiolol in the paediatric population. Due to its unique properties, landiolol allows for a fast onset and offset of heart rate control, flexible weight-based dosing for precise and swift dose adaptation and a minimal impact on blood pressure. These features are of important clinical significance in the treatment of children with SVTs, making landiolol a valuable option for acute situations requiring immediate heart rate control. Landiolol infusion was well tolerated throughout the infusion period, with no novel safety concerns emerging. Based on the efficacy, safety, and PK results, we consider that paediatric patients with SVTs can be successfully treated with landiolol following a similar dosage regimen as in adults. To further refine the therapeutic strategies, more studies are required to complement the existing evidence on the optimal use of pharmacological treatments of SVTs in the paediatric population.

## Supplementary Material

euaf025_Supplementary_Data

## Data Availability

The data sets generated and/or analysed during the current study will not be publicly available to maintain patient confidentiality (but are available from the corresponding author on reasonable request and after the agreement of all the co-authors).
