# Supplementary material for: Landiolol is effective and safe in paediatric supraventricular tachycardia: evidence from a European prospective multicentre open-label phase III study (LANDI-PED)
Source: Europace. 2025 Feb 14;27(2):euaf025. doi: 10.1093/europace/euaf025 (PMC11879349; doi:10.1093/europace/euaf025)

**Supplementary material**

**Supplementary methods:**

**History of protocol amendments**

**All protocol amendments are described below:**

*There were six amendments to the LANDI-PED protocol:*

**Amendment 1:** In agreement with the ethics committee, to minimize the safety risk a staggered enrolment was implemented wherein 5 patients from the oldest age group (>12 years to <18 years) were to be enrolled first, and pending review of the data by the Data Safety Monitoring Board (DSMB), further enrolment of patients in the same group and younger age groups were to be done. Additional DSMB safety reviews could be scheduled at any time, with study enrolment suspended in the event of critical safety concerns.

**Amendment 2:** The exclusion criteria were revised with the addition of “decompensated heart failure”.

**Amendment 3:** The IMP storage conditions, and wording were updated.

**Amendment 4:**  In agreement with PDCO, the staggered enrolment approach was deleted.

**Amendment 5:** The exclusion criteria “treatment with beta-blocker within 12 hours before landiolol start” was deleted. The number of mandatory ECG assessments were reduced to decrease documentation burden.

**Amendment 6:** In agreement with PDCO, sample size was reduced from 120 to 60 patients due to feasibility issues. As per investigators feedback, a more rapid up-titration to next higher dose level after 10 minutes was introduced and reduction of HR to >20% of baseline if medically indicated and deemed safe was allowed. Continuation of landiolol even after conversion to NSR for the entire duration was allowed, if considered safe and medically indicated by the investigator. Exclusion criterion on previous treatment with antiarrhythmic drugs with 24 hours before landiolol infusion start (with the exception of adenosine) was omitted.

Supplement Table S1: List of study sites and investigators in LANDI-PED

| **Country** | **Study site** | **Investigator** |
| --- | --- | --- |
| Austria | Department of Paediatrics and Adolescent Medicine, Division of Paediatric Cardiology, Medical University of Vienna, General Hospital Vienna, Vienna | Prof. Dr. Ina Michel-Behnke |
|  | Clinic for Anaesthesiology and Operative Intensive Care, Kepler University Clinic, Kepler University, Linz | Prof. Dr. Jens Meier |
| Germany | Paediatric Heart Centre Giessen, Department of Anaesthesiology and Intensive Care Medicine, University Hospital Giessen and Marburg GmbH, Giessen | Prof. Dr. Matthias Müller |
|  | Department of Congenital Heart Disease and Paediatric Cardiology, Faculty of Medicine, Heart Centre Freiburg University, University of Freiburg, Freiburg | Prof. Dr. Brigitte Stiller |
|  | Department of Paediatrics and Paediatric Cardiology, Westpfalz Hospital, Kaiserslautern | PD. Dr. Thomas Kriebel |
|  | Department of Paediatric Cardiology, Children’s Heart Centre Aachen, University Hospital RWTH Aachen, Aachen | Dr. Majed Kanaan |
|  | Paediatrics 3 – Centre for Congenital Heart Defects Stuttgart, Paediatric Intensive Care, Pulmonology and Allergology, Stuttgart Hospital – Olgahospital, Stuttgart | Dr. Matthias Mai |
|  | Clinic for Paediatric Cardiology, Heart Centre Leipzig GmbH, Leipzig | Dr. Roman Gebauer |
| Hungary | Gottsegen National Cardiovascular Centre, Paediatric Heart Centre, Budapest | Dr. László Környei |
| Spain | Paediatric Cardiology Department, University Hospital Vall d’ Hebron, Barcelona | Dr. Ferran Roses-Noguer |

**Supplement Table S2: Patient eligibility criteria**

| **Inclusion criteria** | **Exclusion criteria** |
| --- | --- |
| 1. Informed consent/assent from parents/legal representative(s) and children, if applicable.  2. Age: >1 day up to 18 years  3. Body weight at least 2.5 kg.  4. Sustained supraventricular tachyarrhythmia for more than 1 minute.  5. If sub-type paroxysmal supraventricular tachycardia (AVRT, AVNRT) - refractory to treatment with adenosine, relapsed after adenosine treatment and patients with contraindications to adenosine; or other forms of paroxysmal SVT not indicated for adenosine. | 1. Acute cardiogenic shock.  2. Severe, uncorrectable metabolic acidosis.  3. Ventricular tachycardia.  4. Sick sinus syndrome (if there is no possibility for cardiac pacing) or clinically significant bradycardia.  5. Acute asthma.  6. Known pulmonary hypertension.  7. Known stage 4 and 5 chronic renal disease.  8. AV block 2^nd^ or 3^rd^ degree (if there is no possibility for cardiac pacing).  9. Clinically significant hypotension.  10. Postmenstrual age (gestational age + chronological age) <37 weeks.  11. Untreated pheochromocytoma.  12. End-stage disease.  13. Pregnant or breast-feeding patients.  14. Known hypersensitivity to any component of the study medication (e.g. landiolol, mannitol).  15. Participation in a clinical study or exposure to any study medication within 28 days before landiolol infusion start, with the exception of landiolol (end-of-study visit completed).  16. Decompensated heart failure |

**Supplement Figure S1: Dosing scheme for landiolol in the study**

**
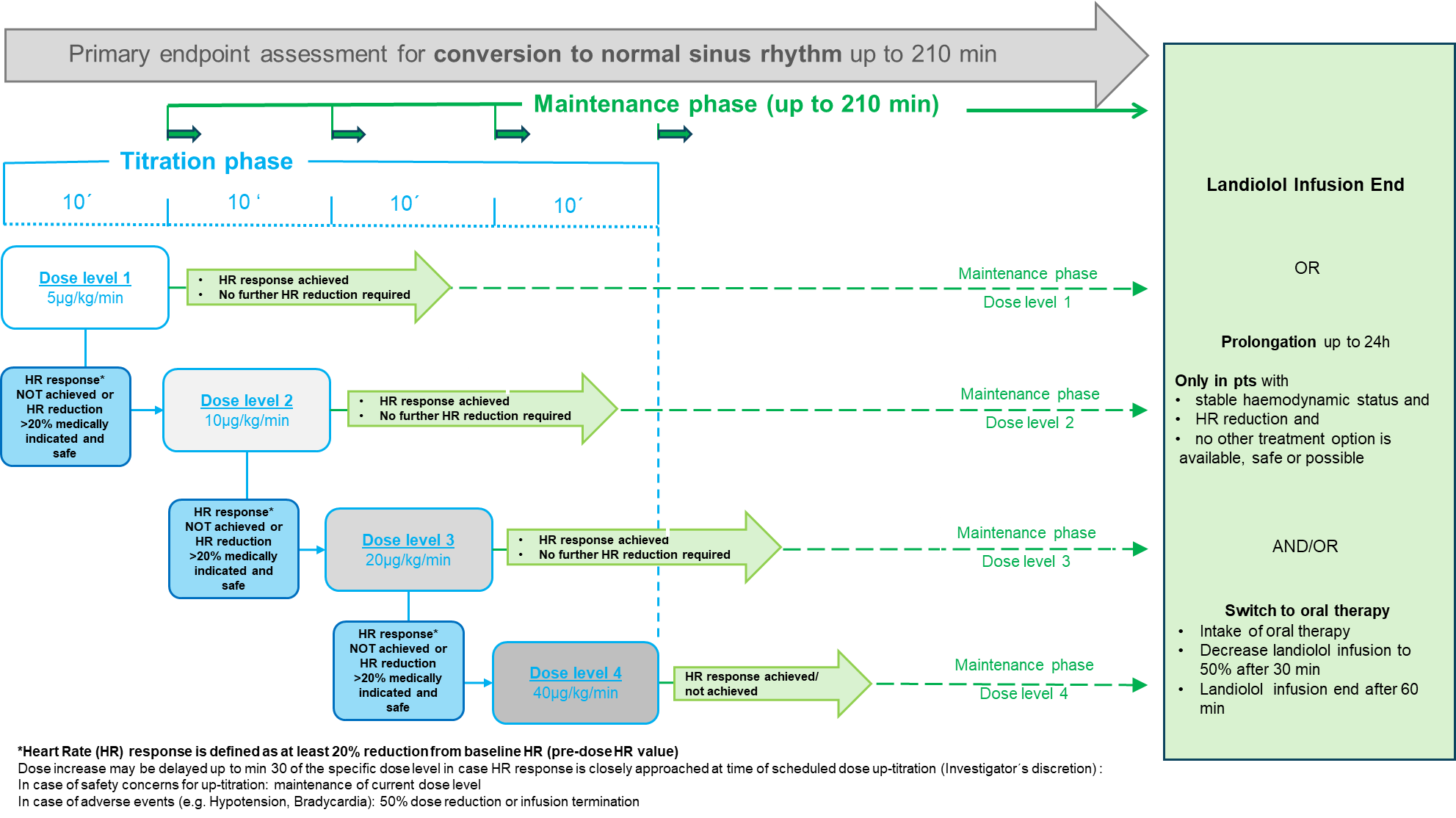
**

**Supplement Figure S2: Schedule of enrolment and assessments (SPIRIT 2013 Figure).**

|  | **Study period** | | | | | | | | |
| --- | --- | --- | --- | --- | --- | --- | --- | --- | --- |
|  | **Enrolment** | **Allocation** | **Post allocation** | | | | | | |
|  |  |  | **Treatment Phase** | | | | **Follow-up Phase** | | |
| **Timepoint** | -T1 |  | T1 | T2 | T3 | T4 | T5 | T6 | T7 |
| **ENROLMENT:** |  |  |  |  |  |  |  |  |  |
| **Eligibility screen** | X |  |  |  |  |  |  |  |  |
| **Informed consent/assent** | X |  |  |  |  |  |  |  |  |
| **Allocation** |  | X |  |  |  |  |  |  |  |
| **INTERVENTION:** |  |  |  |  |  |  |  |  |  |
| **Landiolol infusion as per dosing scheme** |  |  |  |  |  |  |  |  |  |
| **ASSESSMENTS:** |  |  |  |  |  |  |  |  |  |
| **Demographics, Medical history** |  | X |  |  |  |  |  |  |  |
| **Physical examination** |  | X |  |  |  |  |  |  | X |
| **Clinical laboratory** |  | X |  |  |  |  |  | X | X |
| **Vital signs (HR, sBP, dBP), ECG** |  | X | X | X | X | X | X | X | X |
| **PK blood sampling** |  |  |  | X |  | X | X^*^ |  |  |
| **Adverse events** |  |  | X | X | X | X | X | X | X |

-T1: Baseline assessment, T1: 0-30 minutes after landiolol infusion start, T2: 30-210 minutes after landiolol infusion start, T3: 210 minutes – 24 hour after landiolol infusion start, T4: Before landiolol infusion end, T5: 1 hour after landiolol infusion end, T6: 24 hour after landiolol infusion end, T7: 7 days after landiolol infusion end

*Three PK samples were taken immediately after landiolol infusion end (at different timepoints)

Abbreviations: HR - Heart rate; BP - Blood pressure; sBP - Systolic blood pressure; dBP - Diastolic blood pressure; ECG - Electrocardiogram, PK - Pharmacokinetic

Supplement Table S3: Distribution of patients per SVT subtypes and surgical status

|  | **N = 60** | | |
| --- | --- | --- | --- |
| **SVT type at baseline** | **Non-surgical**  **N = 17 (28.3%)** | **Peri-operative**  **N = 14 (23.4%)** | **Post-operative**  **N = 29 (48.3%)** |
| AVRT | 6 (10%) | 0 (0%) | 1 (1.7%) |
| FAT | 6 (10%) | 0 (0%) | 2 (3.3%) |
| IST | 2 (3.3%) | 13 (21.7%) | 15 (25.0%) |
| JET | 2 (3.3%) | 1 (1.7%) | 9 (15.0%) |
| Other | 1 (1.7%) | 0 (0%) | 2 (3.3%) |

Abbreviations: SVT- Supraventricular Tachycardia, IST - Inappropriate Sinus Tachycardia, JET - Junctional Ectopic Tachycardia, FAT - Focal Atrial Tachycardia, AVRT - Atrioventricular Re-entrant Tachycardia.

Data represented as number (percentage)

**Supplement Table S4: Concomitant inotropes and catecholamines administered with landiolol**

| **Medications** | **N=60** |
| --- | --- |
| **Inotropes** |  |
| Milrinone | 30 (50.0%) |
| Levosimendan | 3 (5.0%) |
| Dobutamine | 2 (3.3%) |
| **Catecholamines** |  |
| Norepinephrine | 22 (36.7%) |
| Epinephrine | 6 (10.0%) |
| Phenylephrine | 1 (1.7%) |

Data represented as number (percentage)

**Supplement Figure S3: Kaplan - Meier curve to show time to normal sinus rhythm conversion among responders (n=23). Probability of conversion of NSR is displayed over the entire duration of landiolol infusion.**


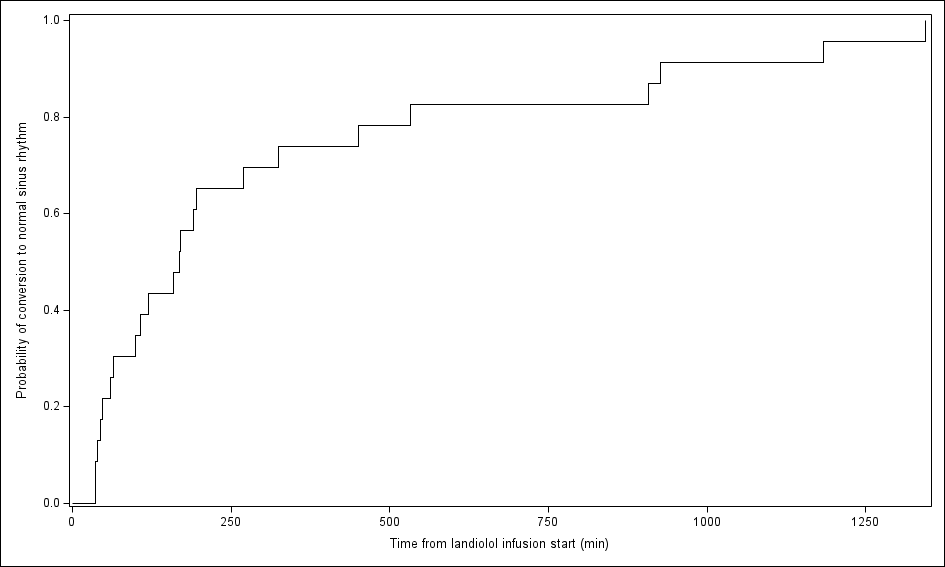


**Supplement Figure S4: Kaplan - Meier curve to show time to achievement of >20% reduction in HR among responders (n=31). Probability of HR response is displayed over the entire duration of landiolol infusion.**


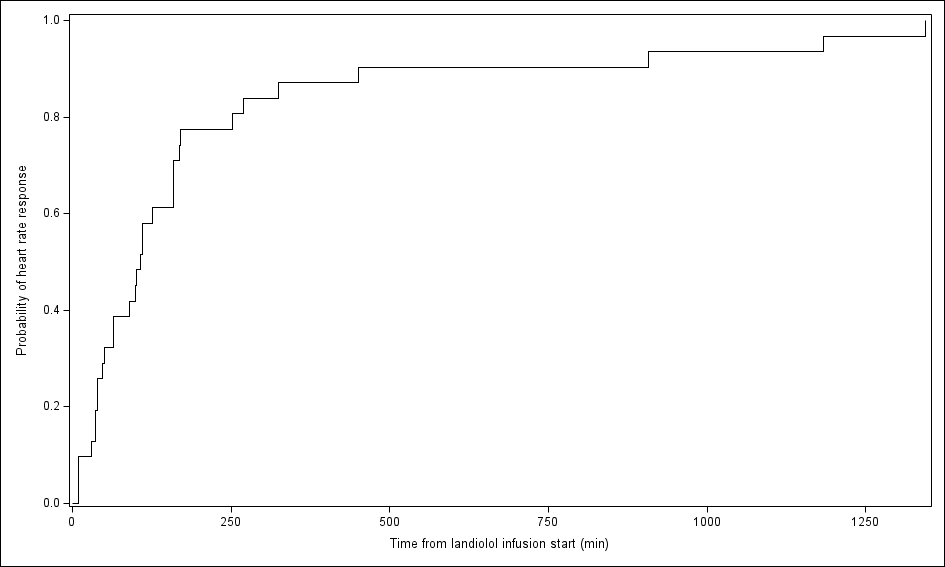


**Supplement Figure S5: Secondary endpoint of percentage reduction in HR from baseline to infusion end and in follow up phase. The change of mean percentage values is displayed.**


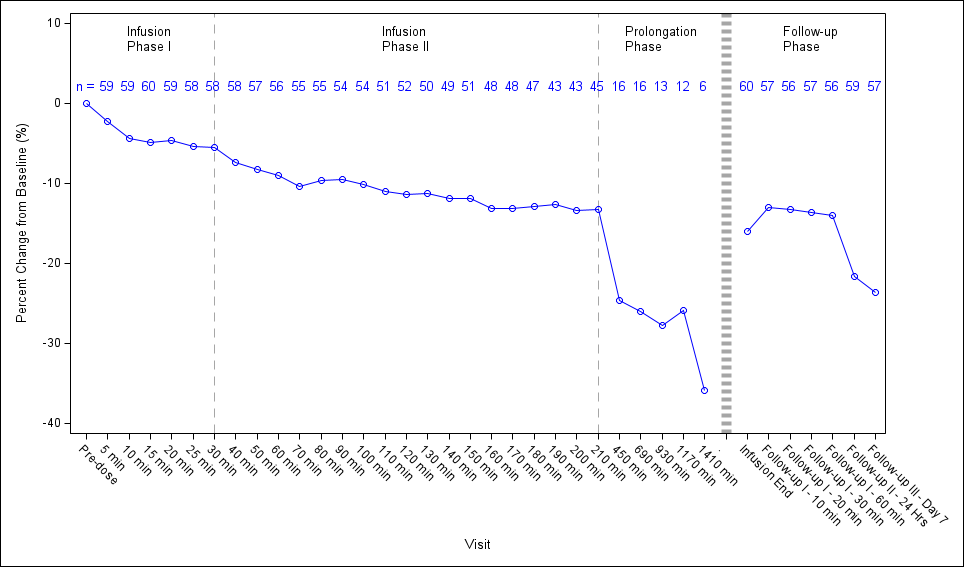


**Supplement Table S5: Duration of landiolol administration and administered dose**

| **Variable** | **Statistics** | **N=60** |
| --- | --- | --- |
| Treatment duration (minutes) | Mean (±SD)  Median (Q1-Q3)  (min-max) | 476.9 (±504.4)  210 (187.5 - 849.5)  (20 – 1440) |
| Total dose (mg/kg) | Mean (±SD)  Median (Q1-Q3)  (min-max) | 14.5 (±17.9)  6.1 (5.8 - 9.4)  (0.1 - 56.8) |
| Maximum dose (µg/kg/min) | Mean (±SD)  Median (Q1-Q3)  (min-max) | 34.9 (±11.1)  40 (40.0 - 40.0)  (5 - 40) |

Data are represented as mean (+SD) and median with (Q1-Q3) and (minimum-maximum)

**Supplement Table S6: Relative and cumulative NSR conversion rates and heart rate response rates at each dosing level**

|  | **Patients with NSR conversion** | | **Patients with ≥ 20% HR reduction** | |
| --- | --- | --- | --- | --- |
| **Landiolol dose** | **Relative response**  **n/N (%)** | **Cumulative response**  **n/N (%)** | **Relative response**  **n/N (%)** | **Cumulative response**  **n/N (%)** |
| 5 µg/kg/min | 0/60 (0.0)  [95% CI: -] | - | 4/60 (6.7)  [95% CI: 0.4; 12.9] | - |
| 10 µg/kg/min | 2/57 (3.5)  [95% CI: 0.0; 8.3] | 2/60 (3.3)  [95% CI: 0.0; 7.9] | 2/57 (3.5)  [95% CI: 0.0; 8.3] | 5/60 (8.3)  [95% CI: 1.3; 15.3] |
| 20 µg/kg/min | 1/53 (1.9)  [95% CI: 0.0; 5.6] | 3/60 (5.0)  [95% CI: 0.0; 10.5] | 6/53 (11.3)  [95% CI: 2.8; 19.9] | 10/60 (16.7)  [95% CI: 7.2; 26.1] |
| 40 µg/kg/min | 19/49 (38.8)  [95% CI: 25.1; 52.4] | 22/60 (36.7)  [95% CI: 24.5; 48.9] | 20/49 (40.8)  [95% CI: 27.1; 54.6] | 29/60 (48.3)  [95% CI: 35.7; 60.9] |
| Overall | - | 23/60 (38.3)  [95% CI: 26.0; 50.6] | - | 31/60 (51.7)  [95% CI: 39.0; 64.3] |

Abbreviations: NSR – Normal Sinus Rhythm, HR – Heart Rate.

Response rates are represented as n= number of responders and N= total number of patients exposed to the indicated dose level with (percentage) and 95% confidence Interval (CI), during landiolol infusion (up to maximum of 24 hours).

**Supplement Figure S6: Changes in HR for up to 210 minutes by patient age. Mean absolute HR (bpm) values (±SD) are displayed.**


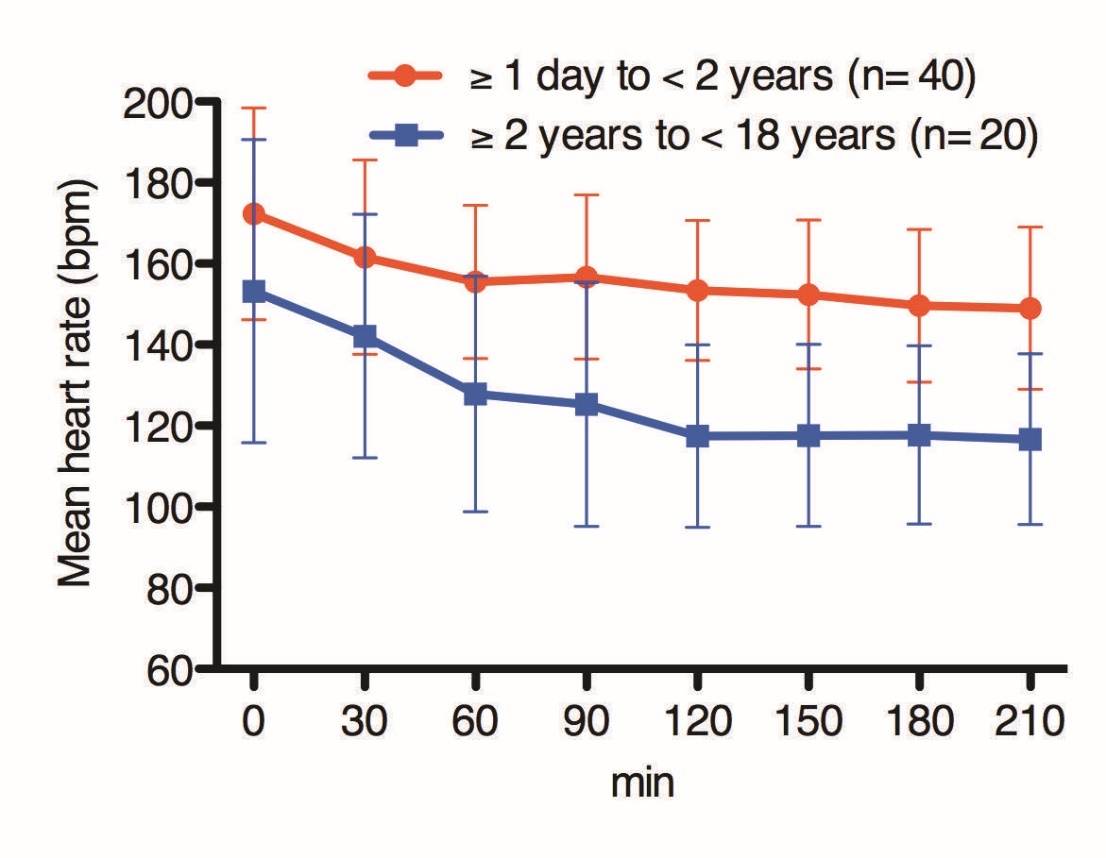

Supplement: euaf025_Supplementary_Data [file euaf025_supplementary_data.docx]
